# Supplementary material for: Remission spectroscopy resolves the mechanism of action of bedaquiline within living mycobacteria
Source: Nat Commun. 2025 Nov 25;16:11018. doi: 10.1038/s41467-025-65928-0 (PMC12695928; doi:10.1038/s41467-025-65928-0)
Supplement: Supplementary file 1 — Supplementary Information [file 41467_2025_65928_MOESM1_ESM.pdf]

Supplementary information for

Remission spectroscopy resolves the mechanism of action of bedaquiline within living mycobacteria

Suzanna H Harrison[1,2*], Rowan C Walters[1,2*], Chen-Yi Cheung[3], Roger J Springett[1,2,4], Gregory M Cook[3,5], Morwan M Osman[1,2*⊥], James N Blaza[1,2⊥]

[1] York Structural Biology Laboratory, Department of Chemistry, University of York, York, YO10 5DD. [2] York Biomedical Research Institute, University of York, York, YO10 5DD. [3] Department of Microbiology and Immunology, University of Otago, Dunedin 9016, New Zealand. [4] Cellspex Ltd, Northamptonshire, UK. [5] School of Biomedical Sciences, Queensland University of Technology, Brisbane, Queensland 4000, Australia.

* These authors contributed equally to the work

⊥ To whom correspondence should be addressed: jamie.blaza@york.ac.uk, morwan.osman@york.ac.uk

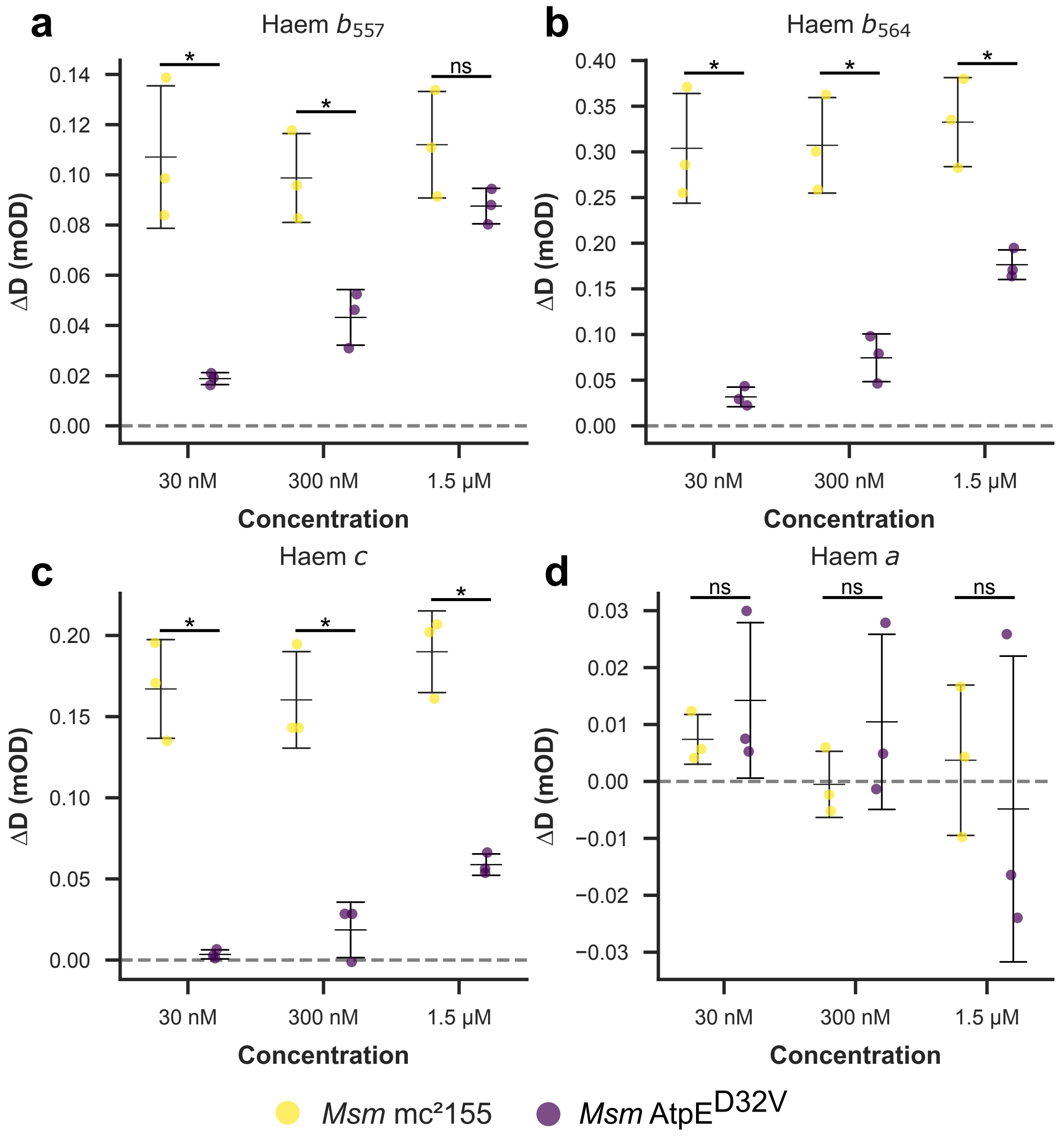

Supplementary Figure 1. Comparison and statistical analysis of cytochrome signals in Fig. 2. Data from (n = 3) independent experiments. 2-tailed Welch's t-test was used with a Bonferroni correction for multiple comparisons. * indicates $p < 0.05$, ns $p \geq 0.05$.

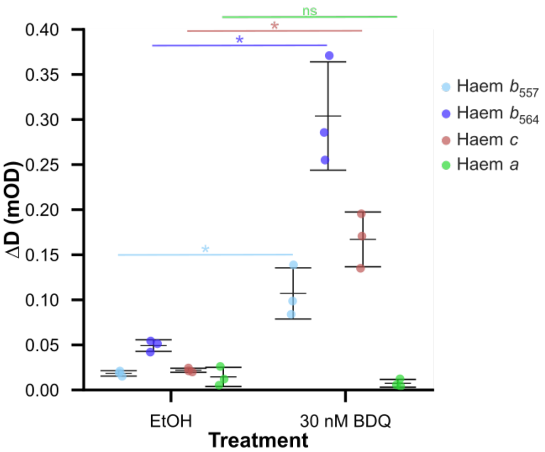

Supplementary Figure 2. Comparison of ethanol (EtOH) and 30 nM bedaquiline (BDQ) dissolved in ethanol in *M. smegmatis*. The bedaquiline (BDQ) response is significantly higher than Ethanol (EtOH) vehicle alone; 5 µL of EtOH was added to the 5mL chamber. Two-tailed Welch's t-test. * indicates $p < 0.05$, ns $p \geq 0.05$.

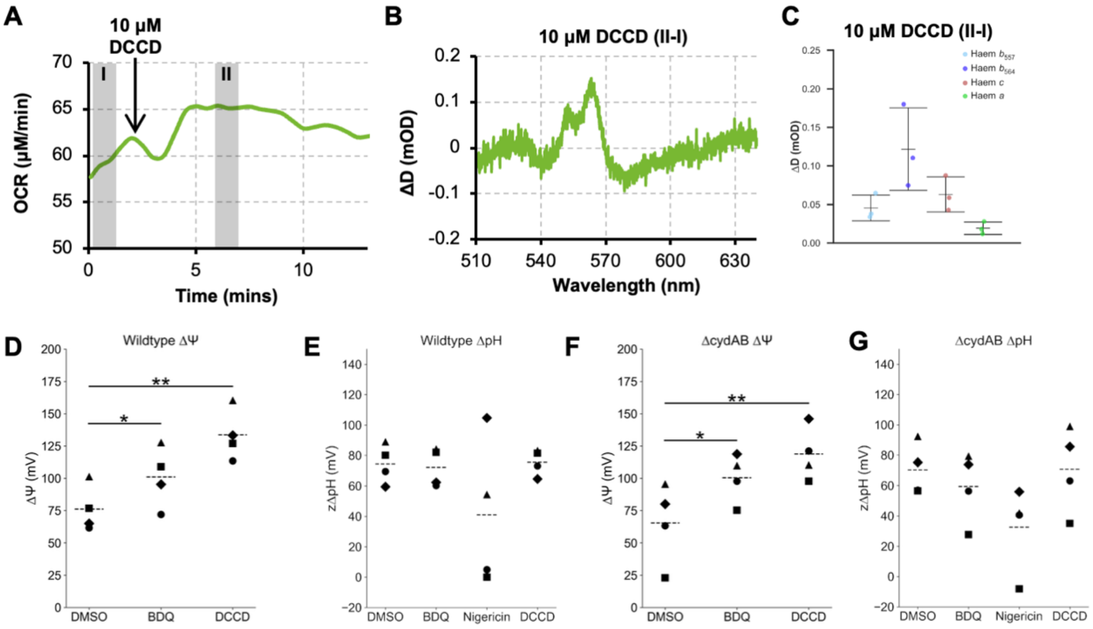

Supplementary Figure 3. Effect of DCCD on *M. smegmatis* and PMF measurements of *M. smegmatis* in response to bedaquiline and DCCD (a-c) OCR traces, remission spectra and changes in individual cytochromes of *M. smegmatis* in response to DCCD, representative traces from $n = 3$ independent experiments. (d-g) Effect of bedaquiline (BDQ) and DCCD on bioenergetic parameters measured with $[^3H]TPP^+$ (ΔΨ) and $[7\text{-}^{14}C]$benzoate (ΔpH). **(c)** Mean is shown ± SD ($n = 3$). **(d)** ΔΨ of *M. smegmatis* after treatment with DMSO, bedaquiline, and DCCD **(e)** ΔpH of *M. smegmatis* after treatment with DMSO, bedaquiline, nigericin, or DCCD **(f)** ΔΨ of *M. smegmatis* Δ*cydAB* after treatment with DMSO, bedaquiline, and DCCD **(g)** ΔpH of *M. smegmatis* Δ*cydAB* after treatment with DMSO, bedaquiline, nigericin, and DCCD. Symbols represent matched biological replicates, ($n = 4$). Dashed lines indicate mean. * indicates p-value < 0.05, **p-value < 0.01. Repeated measures ANOVA with Bonferroni correction for multiple comparisons.

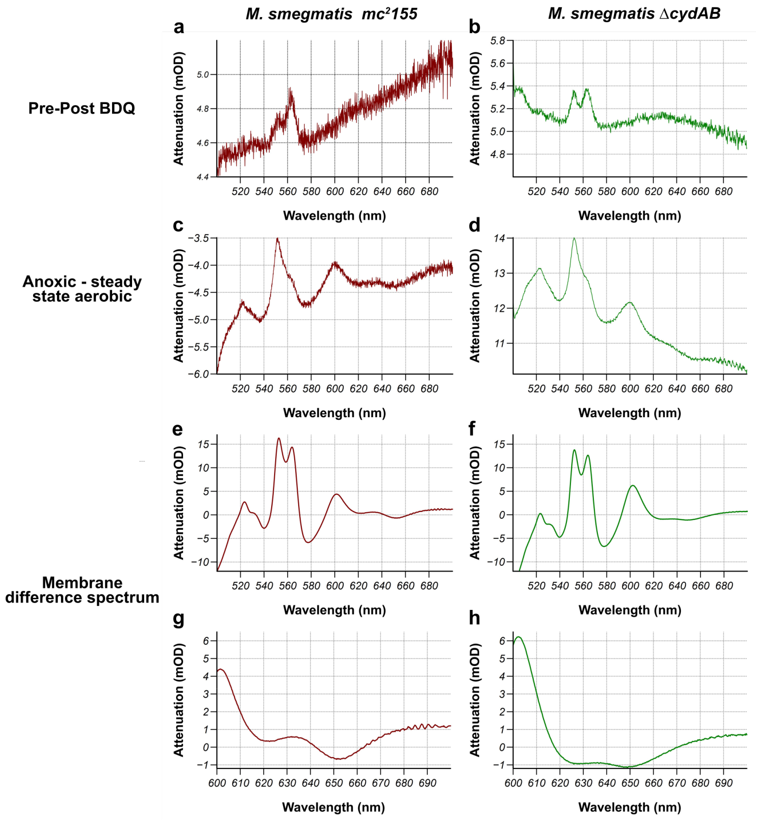

Supplementary Figure 4. Wide-spectra of cells and isolated membranes to detect redox changes in CydAB in WT *M. smegmatis* and Δ*cydAB M. smegmatis*. (a & b) The spectral response to the addition of 30 nM bedaquiline (BDQ) in mc^{2}155 *M. smegmatis* (left) and Δ*cydAB M. smegmatis* (right). Any difference in the region around the cytochrome *a* and cytochrome *d* signals are not above the noise of the spectrum. **(c & d)** Anoxic spectrum minus the aerobic steady state spectrum from mc^{2}155 *M. smegmatis* and Δ*cydAB M. smegmatis*, intact cells. **(e & f)** Reduced-oxidised spectra of mc^{2}155 *M. smegmatis* membranes (e) and Δ*cydAB M. smegmatis* membranes (f). (g & h) Comparison of the cytochrome *d* spectral region of mc^{2}155 and Δ*cydAB*.

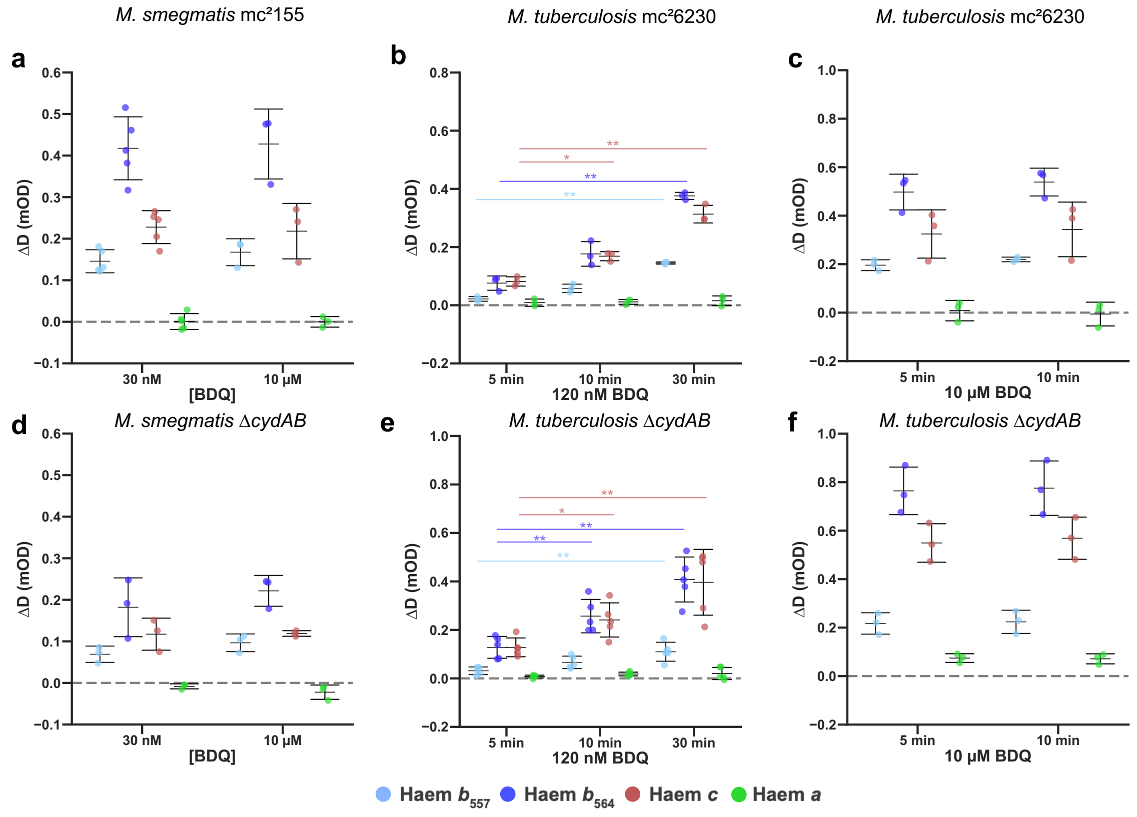

Supplementary Figure 5. Statistical comparisons between datasets in Figure 4. All tests are 2-tailed Welch's t-tests with Bonferroni correction for multiple comparisons. * $p < 0.05$, ** $p < 0.01$. For (a, c, d, and f) no significant difference was found for pairwise comparisons ($p \geq 0.05$).

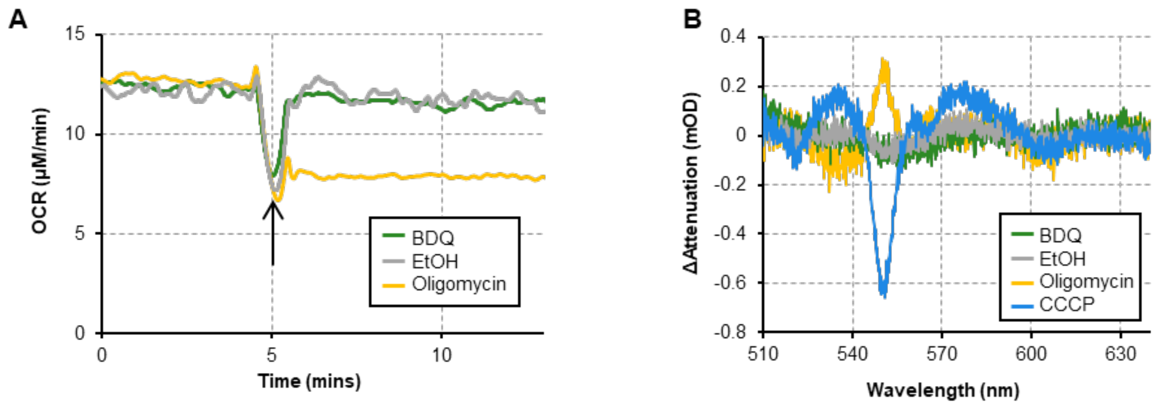

Supplementary Figure 6. Effect of bedaquiline and respiratory inhibitors on the human HEK293T cell line. (A) OCR traces of HEK293T cells treated with 10 µM bedaquiline (BDQ), ethanol vehicle control (EtOH), and 1.5 µM oligomycin A. Arrow indicates the time point at which drug was added. (B) Difference spectra of HEK293T cells treated with 10 µM bedaquiline, ethanol, 1.5 µM oligomycin A, and 1 µM CCCP. Data are representative of n=3 independent experiments.

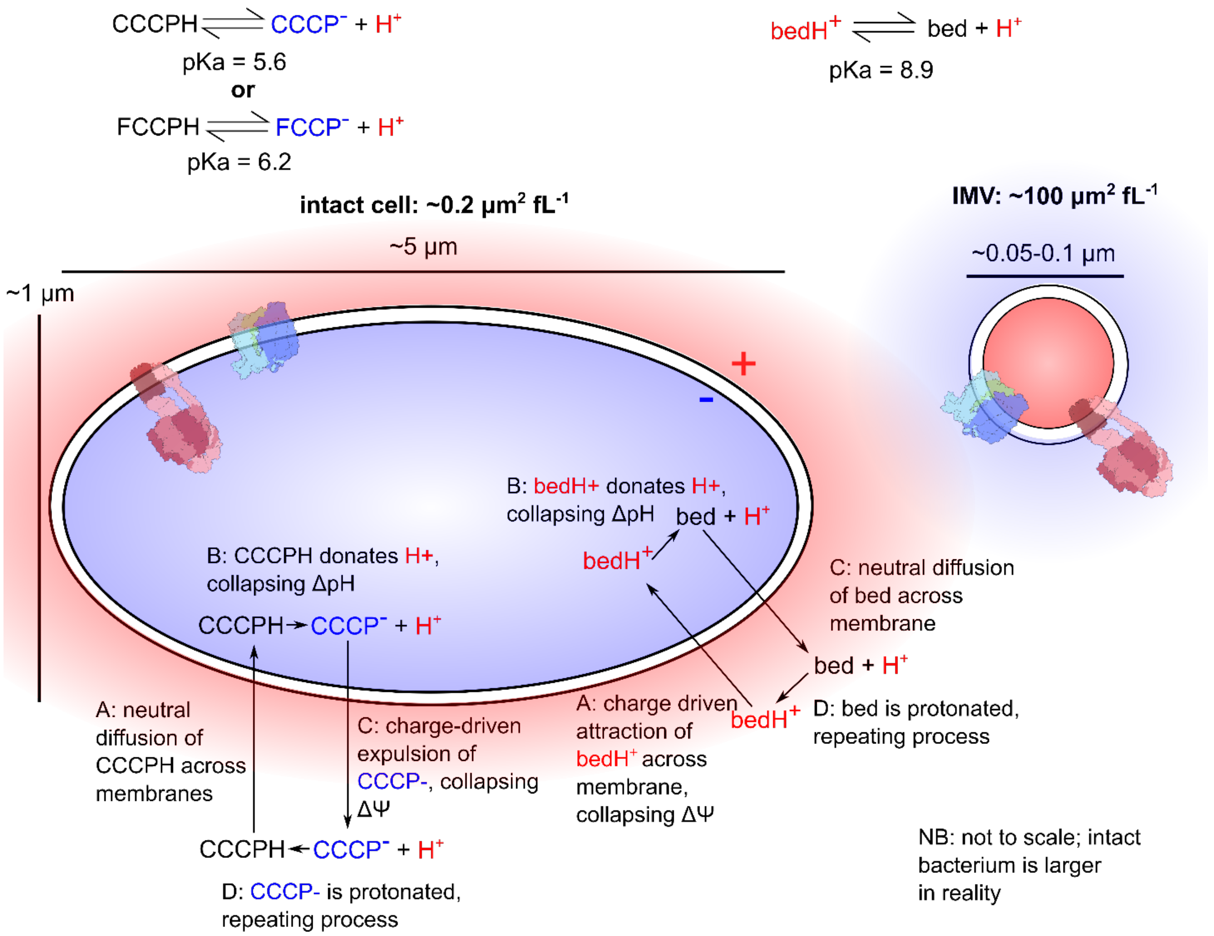

Supplementary Figure 7. Schematic of differences between intact bacteria and IMVs and how this may affect uncoupling by different agents. The polarity of the structures is reversed, with negative/alkali conditions being found inside intact cells and positive/acid conditions being found in the lumen of IMVs. Additionally, the surface area to volume ratio is different; here the ratio was calculated assuming IMVs are spherical and bacteria are cylinders with hemispherical ends. Chemical uncouplers like FCCP and CCCP have neutral protonated forms and negative deprotonated forms, whereas bedaquiline has a positive protonated form and a neutral deprotonated form. These differences may explain why bedaquiline can uncouple IMVs but not intact cells.
